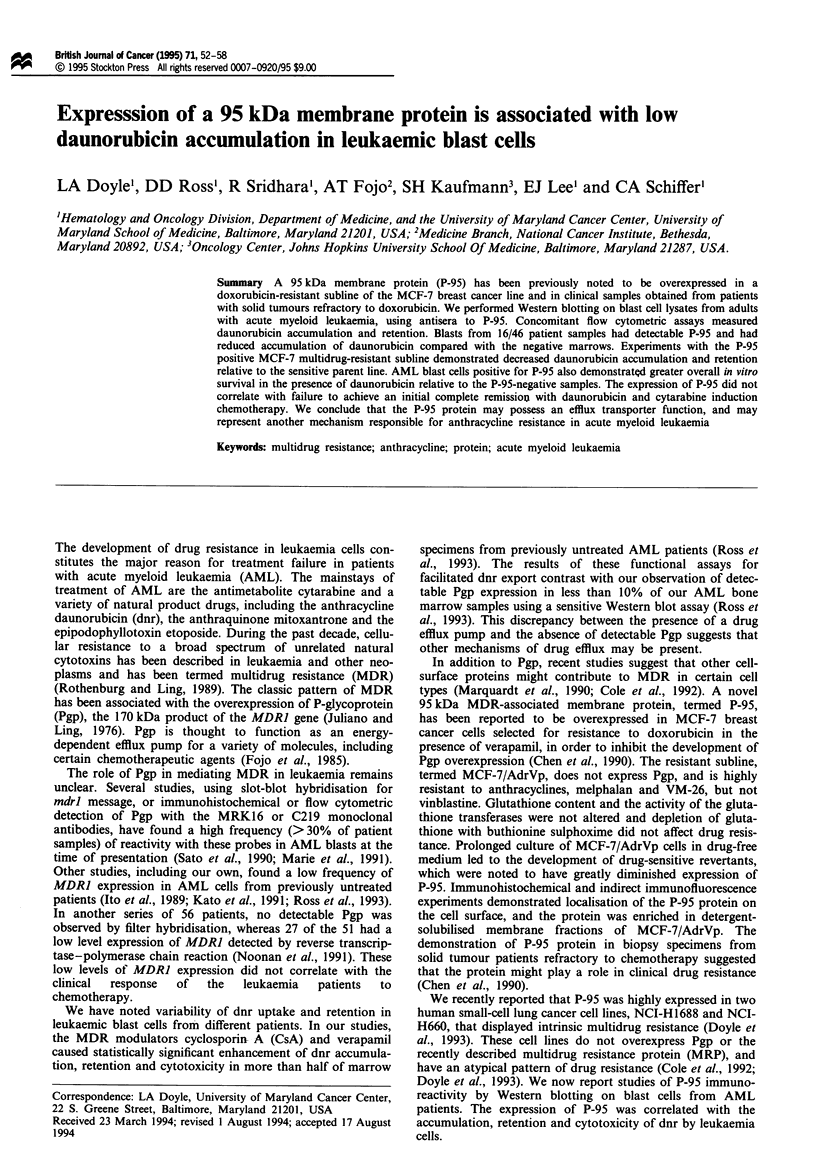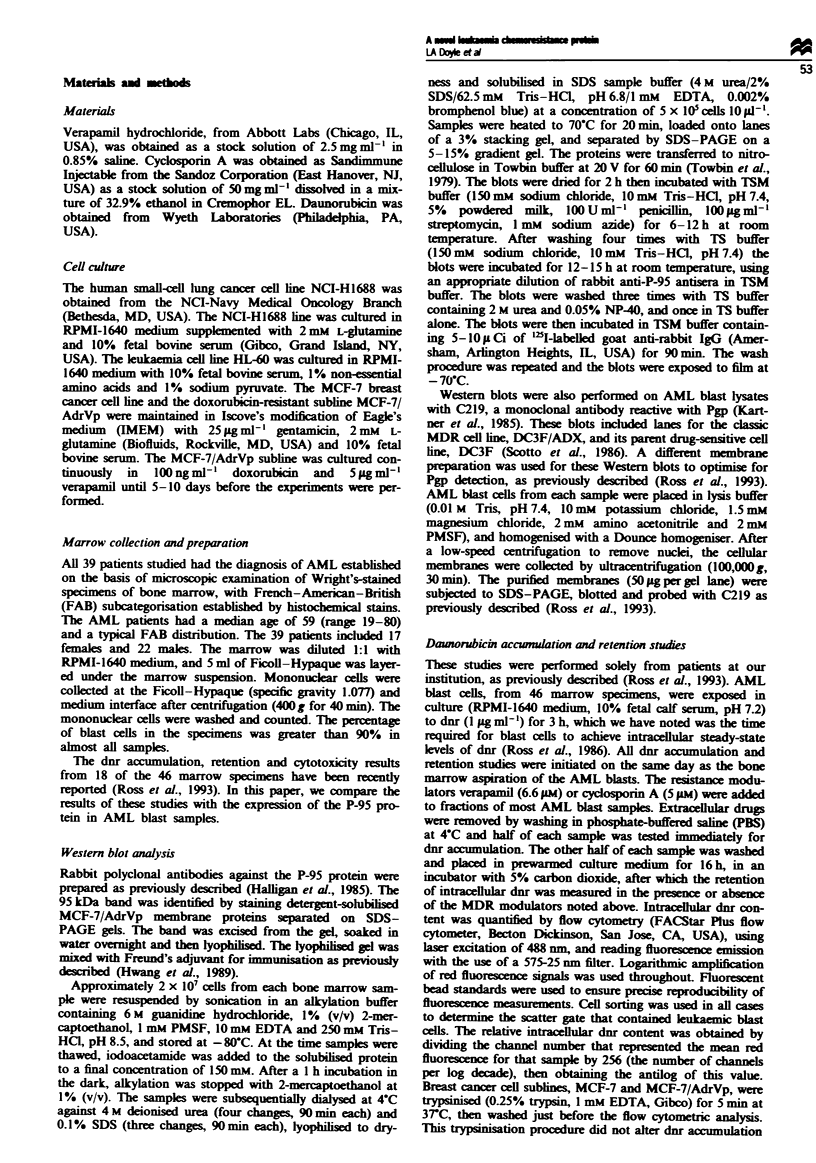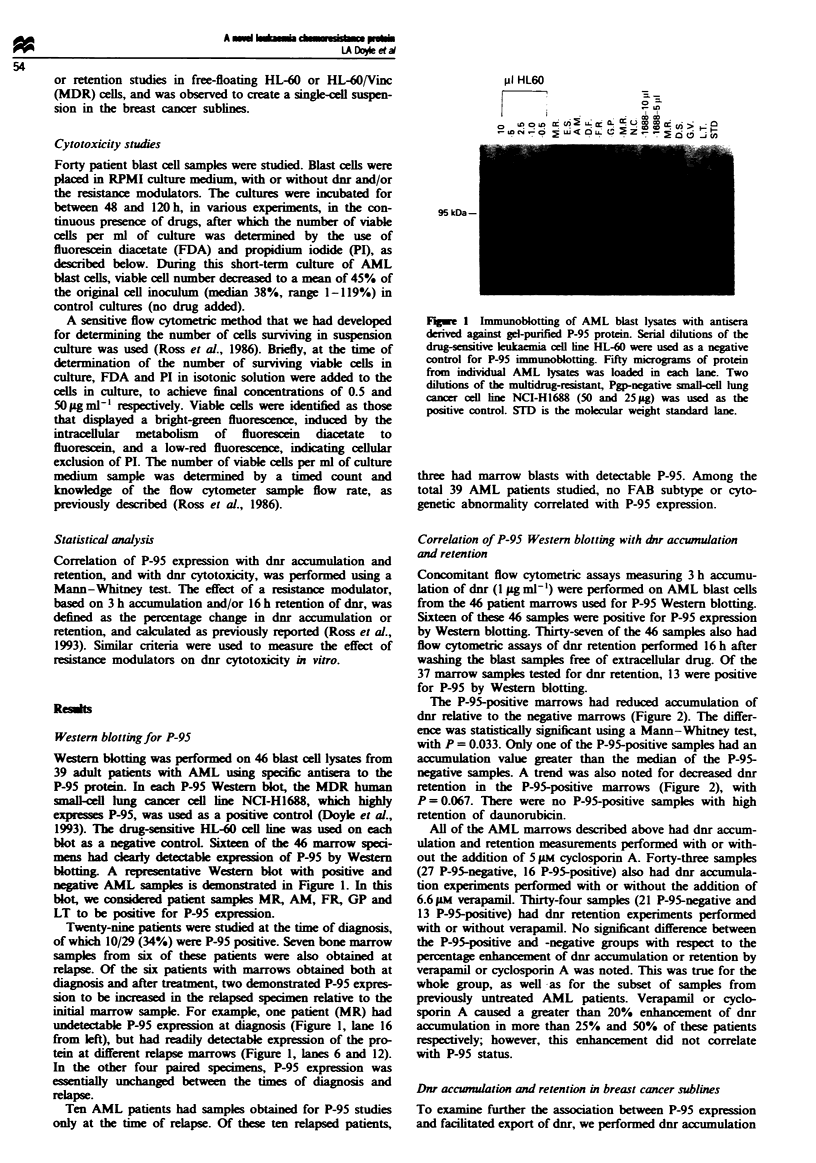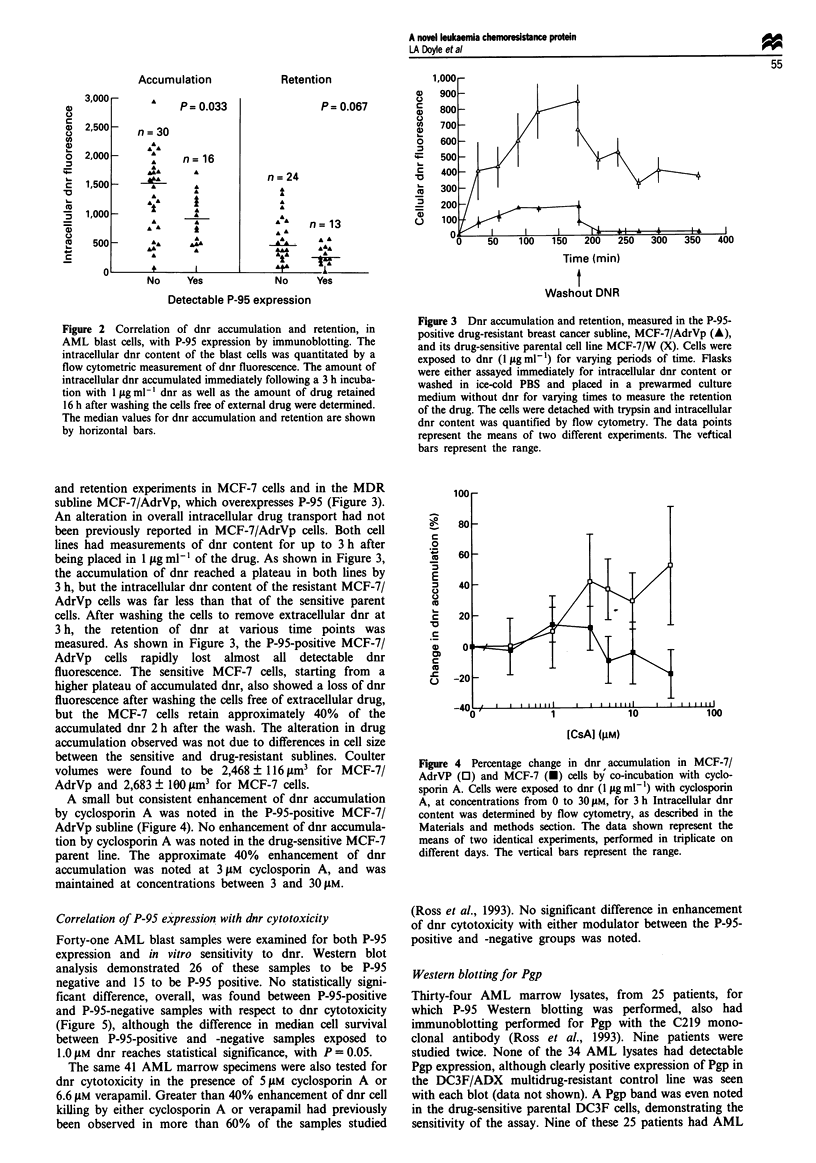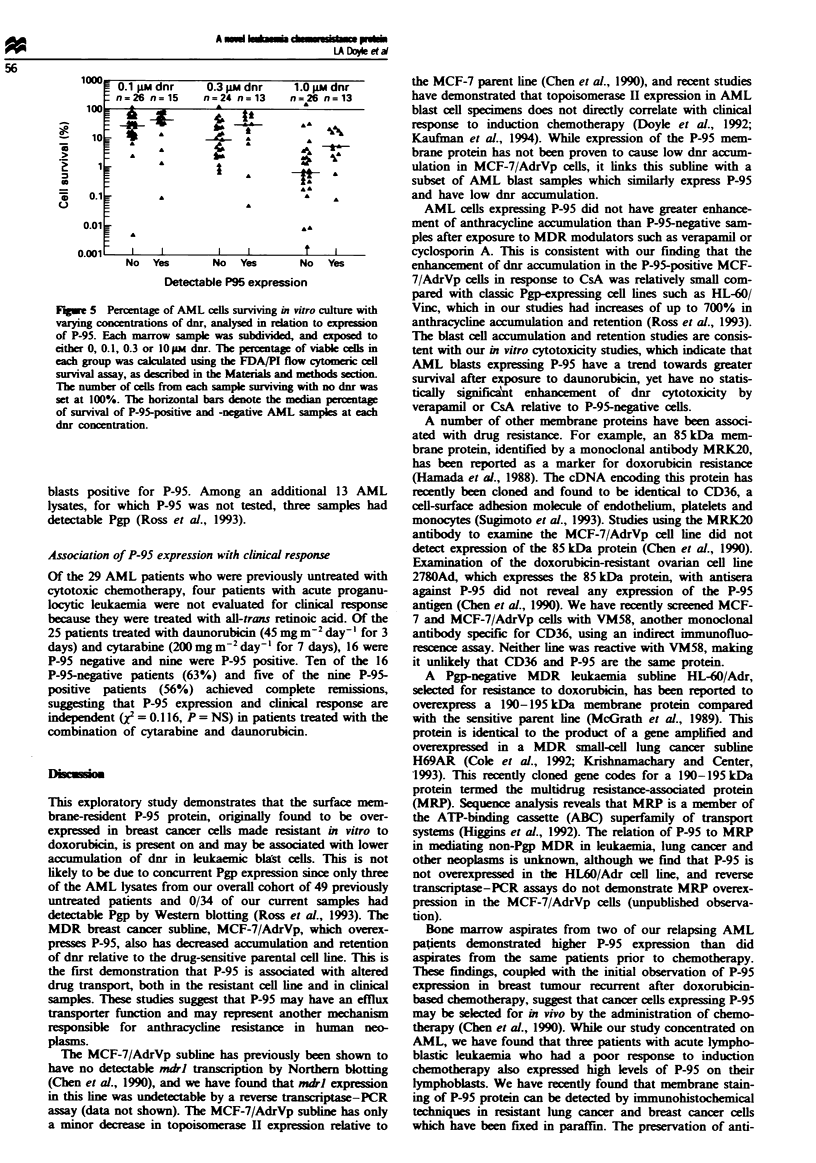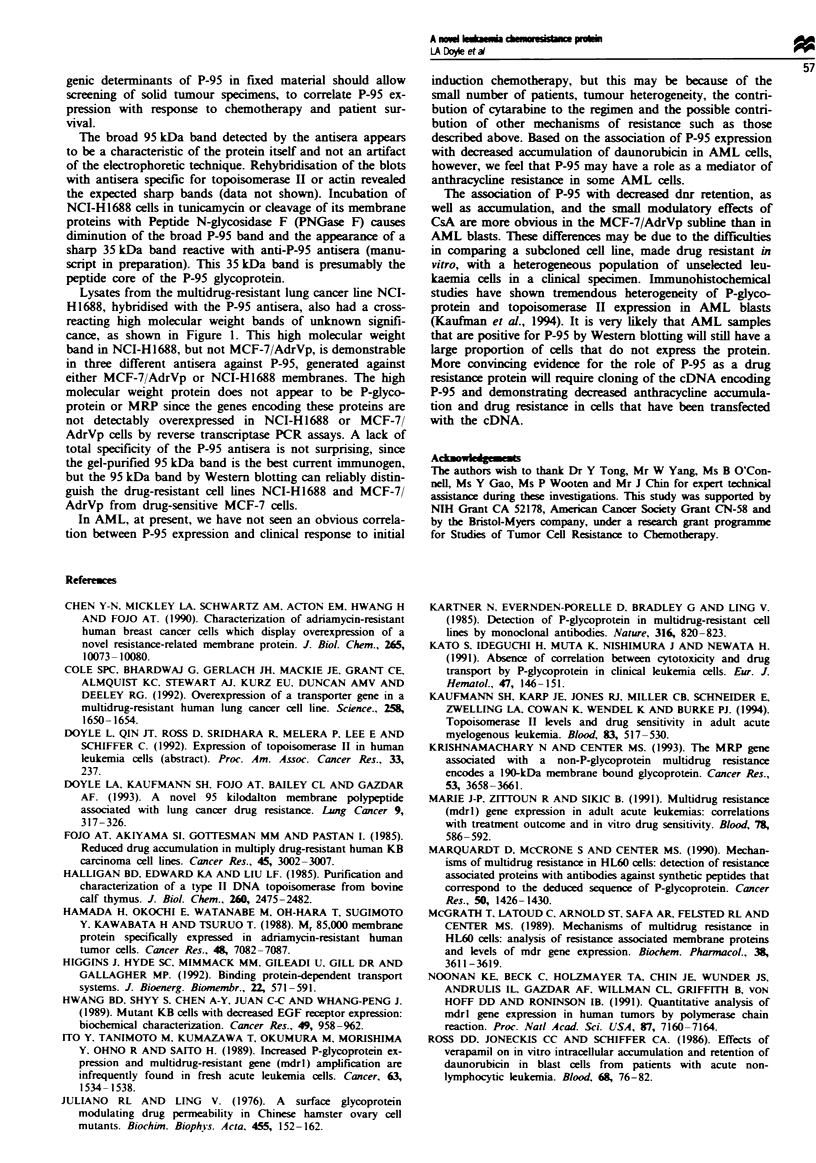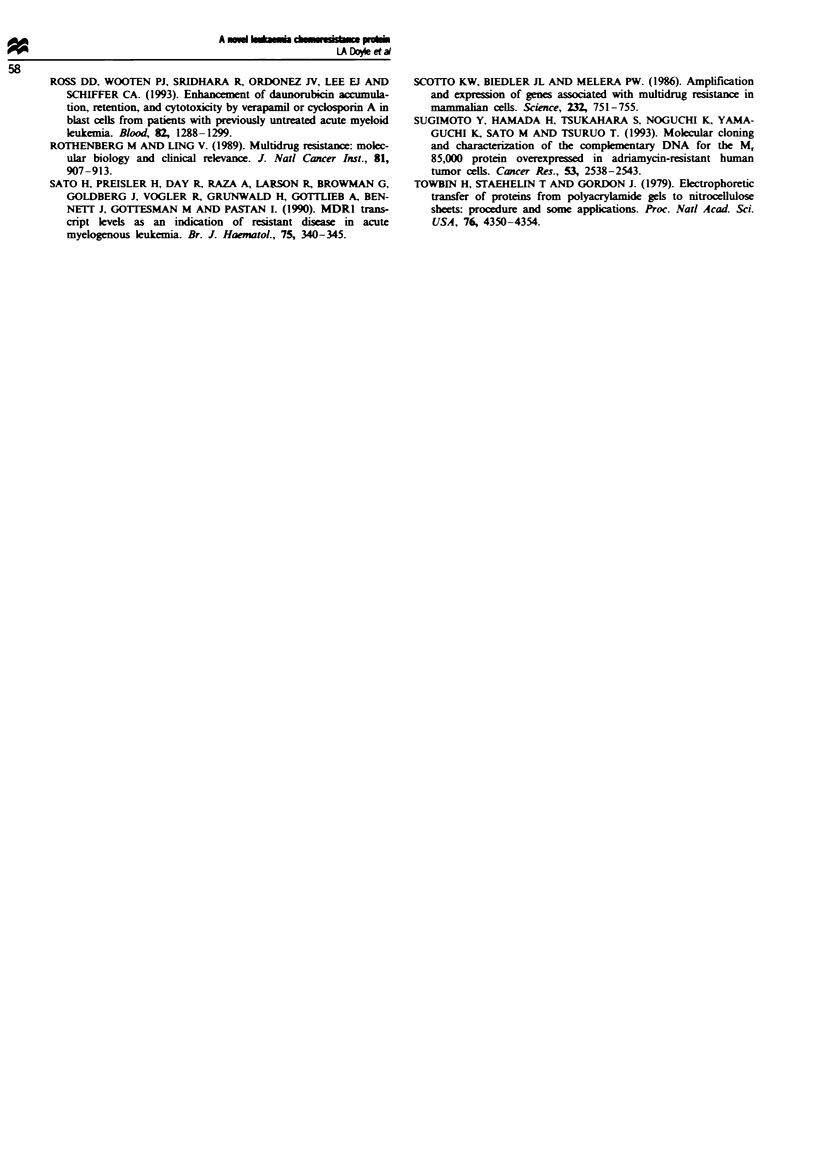# Expression of a 95 kDa membrane protein is associated with low daunorubicin accumulation in leukaemic blast cells.

**DOI:** 10.1038/bjc.1995.11

**Published:** 1995-01

**Authors:** L. A. Doyle, D. D. Ross, R. Sridhara, A. T. Fojo, S. H. Kaufmann, E. J. Lee, C. A. Schiffer

**Affiliations:** Department of Medicine, University of Maryland School of Medicine, Baltimore 21201.

## Abstract

**Images:**


© 1995 Stockton Press All rights reserved 0007-0920/95 $9.00

### Expresssion of a 95 kDa membrane protein is associated with low daunorubicin accumulation in leukaemic blast cells

LA Doyle[1], DD Ross[1], R Sridhara[1], AT Fojo[2], SH Kaufmann[3], EJ Lee[1] and CA Schiffer[1]

[1]*Hematology and Oncology Division, Department of Medicine, and the University of Maryland Cancer Center, University of Maryland School of Medicine, Baltimore, Maryland 21201, USA;* [2]*Medicine Branch, National Cancer Institute, Bethesda, Maryland 20892, USA;* [3]*Oncology Center, Johns Hopkins University School Of Medicine, Baltimore, Maryland 21287, USA.*

**Summary** A 95 kDa membrane protein (P-95) has been previously noted to be overexpressed in a doxorubicin-resistant subline of the MCF-7 breast cancer line and in clinical samples obtained from patients with solid tumours refractory to doxorubicin. We performed Western blotting on blast cell lysates from adults with acute myeloid leukaemia, using antisera to P-95. Concomitant flow cytometric assays measured daunorubicin accumulation and retention. Blasts from 16/46 patient samples had detectable P-95 and had reduced accumulation of daunorubicin compared with the negative marrows. Experiments with the P-95 positive MCF-7 multidrug-resistant subline demonstrated decreased daunorubicin accumulation and retention relative to the sensitive parent line. AML blast cells positive for P-95 also demonstrated greater overall *in vitro* survival in the presence of daunorubicin relative to the P-95-negative samples. The expression of P-95 did not correlate with failure to achieve an initial complete remission with daunorubicin and cytarabine induction chemotherapy. We conclude that the P-95 protein may possess an efflux transporter function, and may represent another mechanism responsible for anthracycline resistance in acute myeloid leukaemia

**Keywords:** multidrug resistance; anthracycline; protein; acute myeloid leukaemia

The development of drug resistance in leukaemia cells constitutes the major reason for treatment failure in patients with acute myeloid leukaemia (AML). The mainstays of treatment of AML are the antimetabolite cytarabine and a variety of natural product drugs, including the anthracycline daunorubicin (dnr), the anthraquinone mitoxantrone and the epipodophyllotoxin etoposide. During the past decade, cellular resistance to a broad spectrum of unrelated natural cytotoxins has been described in leukaemia and other neoplasms and has been termed multidrug resistance (MDR) (Rothenburg and Ling, 1989). The classic pattern of MDR has been associated with the overexpression of P-glycoprotein (Pgp), the 170 kDa product of the *MDR1* gene (Juliano and Ling, 1976). Pgp is thought to function as an energy-dependent efflux pump for a variety of molecules, including certain chemotherapeutic agents (Fojo *et al.*, 1985).

The role of Pgp in mediating MDR in leukaemia remains unclear. Several studies, using slot-blot hybridisation for *mdr1* message, or immunohistochemical or flow cytometric detection of Pgp with the MRK16 or C219 monoclonal antibodies, have found a high frequency (>30% of patient samples) of reactivity with these probes in AML blasts at the time of presentation (Sato *et al.*, 1990; Marie *et al.*, 1991). Other studies, including our own, found a low frequency of *MDR1* expression in AML cells from previously untreated patients (Ito *et al.*, 1989; Kato *et al.*, 1991; Ross *et al.*, 1993). In another series of 56 patients, no detectable Pgp was observed by filter hybridisation, whereas 27 of the 51 had a low level expression of *MDR1* detected by reverse transcriptase–polymerase chain reaction (Noonan *et al.*, 1991). These low levels of *MDR1* expression did not correlate with the clinical response of the leukaemia patients to chemotherapy.

We have noted variability of dnr uptake and retention in leukaemic blast cells from different patients. In our studies, the MDR modulators cyclosporin A (CsA) and verapamil caused statistically significant enhancement of dnr accumulation, retention and cytotoxicity in more than half of marrow specimens from previously untreated AML patients (Ross *et al.*, 1993). The results of these functional assays for facilitated dnr export contrast with our observation of detectable Pgp expression in less than 10% of our AML bone marrow samples using a sensitive Western blot assay (Ross *et al.*, 1993). This discrepancy between the presence of a drug efflux pump and the absence of detectable Pgp suggests that other mechanisms of drug efflux may be present.

In addition to Pgp, recent studies suggest that other cell-surface proteins might contribute to MDR in certain cell types (Marquardt *et al.*, 1990; Cole *et al.*, 1992). A novel 95 kDa MDR-associated membrane protein, termed P-95, has been reported to be overexpressed in MCF-7 breast cancer cells selected for resistance to doxorubicin in the presence of verapamil, in order to inhibit the development of Pgp overexpression (Chen *et al.*, 1990). The resistant subline, termed MCF-7/AdrVp, does not express Pgp, and is highly resistant to anthracyclines, melphalan and VM-26, but not vinblastine. Glutathione content and the activity of the glutathione transferases were not altered and depletion of glutathione with buthionine sulphoxime did not affect drug resistance. Prolonged culture of MCF-7/AdrVp cells in drug-free medium led to the development of drug-sensitive revertants, which were noted to have greatly diminished expression of P-95. Immunohistochemical and indirect immunofluorescence experiments demonstrated localisation of the P-95 protein on the cell surface, and the protein was enriched in detergent-solubilised membrane fractions of MCF-7/AdrVp. The demonstration of P-95 protein in biopsy specimens from solid tumour patients refractory to chemotherapy suggested that the protein might play a role in clinical drug resistance (Chen *et al.*, 1990).

We recently reported that P-95 was highly expressed in two human small-cell lung cancer cell lines, NCI-H1688 and NCI-H660, that displayed intrinsic multidrug resistance (Doyle *et al.*, 1993). These cell lines do not overexpress Pgp or the recently described multidrug resistance protein (MRP), and have an atypical pattern of drug resistance (Cole *et al.*, 1992; Doyle *et al.*, 1993). We now report studies of P-95 immunoreactivity by Western blotting on blast cells from AML patients. The expression of P-95 was correlated with the accumulation, retention and cytotoxicity of dnr by leukaemia cells.

Correspondence: LA Doyle, University of Maryland Cancer Center, 22 S. Greene Street, Baltimore, Maryland 21201, USA
Received 23 March 1994; revised 1 August 1994; accepted 17 August 1994

### Materials and methods

### *Materials*

Verapamil hydrochloride, from Abbott Labs (Chicago, IL, USA), was obtained as a stock solution of 2.5 mg $ml^{-1}$ in 0.85% saline. Cyclosporin A was obtained as Sandimmune Injectable from the Sandoz Corporation (East Hanover, NJ, USA) as a stock solution of 50 mg $ml^{-1}$ dissolved in a mixture of 32.9% ethanol in Cremophor EL. Daunorubicin was obtained from Wyeth Laboratories (Philadelphia, PA, USA).

### *Cell culture*

The human small-cell lung cancer cell line NCI-H1688 was obtained from the NCI-Navy Medical Oncology Branch (Bethesda, MD, USA). The NCI-H1688 line was cultured in RPMI-1640 medium supplemented with 2 mM L-glutamine and 10% fetal bovine serum (Gibco, Grand Island, NY, USA). The leukaemia cell line HL-60 was cultured in RPMI-1640 medium with 10% fetal bovine serum, 1% non-essential amino acids and 1% sodium pyruvate. The MCF-7 breast cancer cell line and the doxorubicin-resistant subline MCF-7/AdrVp were maintained in Iscove's modification of Eagle's medium (IMEM) with 25 μg $ml^{-1}$ gentamicin, 2 mM L-glutamine (Biofluids, Rockville, MD, USA) and 10% fetal bovine serum. The MCF-7/AdrVp subline was cultured continuously in 100 ng $ml^{-1}$ doxorubicin and 5 μg $ml^{-1}$ verapamil until 5–10 days before the experiments were performed.

### *Marrow collection and preparation*

All 39 patients studied had the diagnosis of AML established on the basis of microscopic examination of Wright's-stained specimens of bone marrow, with French–American–British (FAB) subcategorisation established by histochemical stains. The AML patients had a median age of 59 (range 19–80) and a typical FAB distribution. The 39 patients included 17 females and 22 males. The marrow was diluted 1:1 with RPMI-1640 medium, and 5 ml of Ficoll–Hypaque was layered under the marrow suspension. Mononuclear cells were collected at the Ficoll–Hypaque (specific gravity 1.077) and medium interface after centrifugation (400 *g* for 40 min). The mononuclear cells were washed and counted. The percentage of blast cells in the specimens was greater than 90% in almost all samples.

The dnr accumulation, retention and cytotoxicity results from 18 of the 46 marrow specimens have been recently reported (Ross *et al.*, 1993). In this paper, we compare the results of these studies with the expression of the P-95 protein in AML blast samples.

### *Western blot analysis*

Rabbit polyclonal antibodies against the P-95 protein were prepared as previously described (Halligan *et al.*, 1985). The 95 kDa band was identified by staining detergent-solubilised MCF-7/AdrVp membrane proteins separated on SDS-PAGE gels. The band was excised from the gel, soaked in water overnight and then lyophilised. The lyophilised gel was mixed with Freund's adjuvant for immunisation as previously described (Hwang *et al.*, 1989).

Approximately $2 \times 10^7$ cells from each bone marrow sample were resuspended by sonication in an alkylation buffer containing 6 M guanidine hydrochloride, 1% (v/v) 2-mercaptoethanol, 1 mM PMSF, 10 mM EDTA and 250 mM Tris–HCl, pH 8.5, and stored at −80°C. At the time samples were thawed, iodoacetamide was added to the solubilised protein to a final concentration of 150 mM. After a 1 h incubation in the dark, alkylation was stopped with 2-mercaptoethanol at 1% (v/v). The samples were subsequentially dialysed at 4°C against 4 M deionised urea (four changes, 90 min each) and 0.1% SDS (three changes, 90 min each), lyophilised to dryness and solubilised in SDS sample buffer (4 M urea/2% SDS/62.5 mM Tris–HCl, pH 6.8/1 mM EDTA, 0.002% bromphenol blue) at a concentration of $5 \times 10^5$ cells 10 $\mu l^{-1}$. Samples were heated to 70°C for 20 min, loaded onto lanes of a 3% stacking gel, and separated by SDS–PAGE on a 5–15% gradient gel. The proteins were transferred to nitrocellulose in Towbin buffer at 20 V for 60 min (Towbin *et al.*, 1979). The blots were dried for 2 h then incubated with TSM buffer (150 mM sodium chloride, 10 mM Tris–HCl, pH 7.4, 5% powdered milk, 100 U $ml^{-1}$ penicillin, 100 μg $ml^{-1}$ streptomycin, 1 mM sodium azide) for 6–12 h at room temperature. After washing four times with TS buffer (150 mM sodium chloride, 10 mM Tris–HCl, pH 7.4) the blots were incubated for 12–15 h at room temperature, using an appropriate dilution of rabbit anti-P-95 antisera in TSM buffer. The blots were washed three times with TS buffer containing 2 M urea and 0.05% NP-40, and once in TS buffer alone. The blots were then incubated in TSM buffer containing 5–10 μCi of $^{125}I$-labelled goat anti-rabbit IgG (Amersham, Arlington Heights, IL, USA) for 90 min. The wash procedure was repeated and the blots were exposed to film at −70°C.

Western blots were also performed on AML blast lysates with C219, a monoclonal antibody reactive with Pgp (Kartner *et al.*, 1985). These blots included lanes for the classic MDR cell line, DC3F/ADX, and its parent drug-sensitive cell line, DC3F (Scotto *et al.*, 1986). A different membrane preparation was used for these Western blots to optimise for Pgp detection, as previously described (Ross *et al.*, 1993). AML blast cells from each sample were placed in lysis buffer (0.01 M Tris, pH 7.4, 10 mM potassium chloride, 1.5 mM magnesium chloride, 2 mM amino acetonitrile and 2 mM PMSF), and homogenised with a Dounce homogeniser. After a low-speed centrifugation to remove nuclei, the cellular membranes were collected by ultracentrifugation (100,000 *g*, 30 min). The purified membranes (50 μg per gel lane) were subjected to SDS–PAGE, blotted and probed with C219 as previously described (Ross *et al.*, 1993).

### *Daunorubicin accumulation and retention studies*

These studies were performed solely from patients at our institution, as previously described (Ross *et al.*, 1993). AML blast cells, from 46 marrow specimens, were exposed in culture (RPMI-1640 medium, 10% fetal calf serum, pH 7.2) to dnr (1 μg $ml^{-1}$) for 3 h, which we have noted was the time required for blast cells to achieve intracellular steady-state levels of dnr (Ross *et al.*, 1986). All dnr accumulation and retention studies were initiated on the same day as the bone marrow aspiration of the AML blasts. The resistance modulators verapamil (6.6 μM) or cyclosporin A (5 μM) were added to fractions of most AML blast samples. Extracellular drugs were removed by washing in phosphate-buffered saline (PBS) at 4°C and half of each sample was tested immediately for dnr accumulation. The other half of each sample was washed and placed in prewarmed culture medium for 16 h, in an incubator with 5% carbon dioxide, after which the retention of intracellular dnr was measured in the presence or absence of the MDR modulators noted above. Intracellular dnr content was quantified by flow cytometry (FACStar Plus flow cytometer, Becton Dickinson, San Jose, CA, USA), using laser excitation of 488 nm, and reading fluorescence emission with the use of a 575-25 nm filter. Logarithmic amplification of red fluorescence signals was used throughout. Fluorescent bead standards were used to ensure precise reproducibility of fluorescence measurements. Cell sorting was used in all cases to determine the scatter gate that contained leukaemic blast cells. The relative intracellular dnr content was obtained by dividing the channel number that represented the mean red fluorescence for that sample by 256 (the number of channels per log decade), then obtaining the antilog of this value. Breast cancer cell sublines, MCF-7 and MCF-7/AdrVp, were trypsinised (0.25% trypsin, 1 mM EDTA, Gibco) for 5 min at 37°C, then washed just before the flow cytometric analysis. This trypsinisation procedure did not alter dnr accumulation or retention studies in free-floating HL-60 or HL-60/Vinc (MDR) cells, and was observed to create a single-cell suspension in the breast cancer sublines.

*Cytotoxicity studies*

Forty patient blast cell samples were studied. Blast cells were placed in RPMI culture medium, with or without dnr and/or the resistance modulators. The cultures were incubated for between 48 and 120 h, in various experiments, in the continuous presence of drugs, after which the number of viable cells per ml of culture was determined by the use of fluorescein diacetate (FDA) and propidium iodide (PI), as described below. During this short-term culture of AML blast cells, viable cell number decreased to a mean of 45% of the original cell inoculum (median 38%, range 1–119%) in control cultures (no drug added).

A sensitive flow cytometric method that we had developed for determining the number of cells surviving in suspension culture was used (Ross *et al*., 1986). Briefly, at the time of determination of the number of surviving viable cells in culture, FDA and PI in isotonic solution were added to the cells in culture, to achieve final concentrations of 0.5 and 50 $\mu$g ml$^{-1}$ respectively. Viable cells were identified as those that displayed a bright-green fluorescence, induced by the intracellular metabolism of fluorescein diacetate to fluorescein, and a low-red fluorescence, indicating cellular exclusion of PI. The number of viable cells per ml of culture medium sample was determined by a timed count and knowledge of the flow cytometer sample flow rate, as previously described (Ross *et al*., 1986).

*Statistical analysis*

Correlation of P-95 expression with dnr accumulation and retention, and with dnr cytotoxicity, was performed using a Mann–Whitney test. The effect of a resistance modulator, based on 3 h accumulation and/or 16 h retention of dnr, was defined as the percentage change in dnr accumulation or retention, and calculated as previously reported (Ross *et al*., 1993). Similar criteria were used to measure the effect of resistance modulators on dnr cytotoxicity *in vitro*.

### Results

*Western blotting for P-95*

Western blotting was performed on 46 blast cell lysates from 39 adult patients with AML using specific antisera to the P-95 protein. In each P-95 Western blot, the MDR human small-cell lung cancer cell line NCI-H1688, which highly expresses P-95, was used as a positive control (Doyle *et al*., 1993). The drug-sensitive HL-60 cell line was used on each blot as a negative control. Sixteen of the 46 marrow specimens had clearly detectable expression of P-95 by Western blotting. A representative Western blot with positive and negative AML samples is demonstrated in Figure 1. In this blot, we considered patient samples MR, AM, FR, GP and LT to be positive for P-95 expression.

Twenty-nine patients were studied at the time of diagnosis, of which 10/29 (34%) were P-95 positive. Seven bone marrow samples from six of these patients were also obtained at relapse. Of the six patients with marrows obtained both at diagnosis and after treatment, two demonstrated P-95 expression to be increased in the relapsed specimen relative to the initial marrow sample. For example, one patient (MR) had undetectable P-95 expression at diagnosis (Figure 1, lane 16 from left), but had readily detectable expression of the protein at different relapse marrows (Figure 1, lanes 6 and 12). In the other four paired specimens, P-95 expression was essentially unchanged between the times of diagnosis and relapse.

Ten AML patients had samples obtained for P-95 studies only at the time of relapse. Of these ten relapsed patients,

**Figure 1** Immunoblotting of AML blast lysates with antisera derived against gel-purified P-95 protein. Serial dilutions of the drug-sensitive leukaemia cell line HL-60 were used as a negative control for P-95 immunoblotting. Fifty micrograms of protein from individual AML lysates was loaded in each lane. Two dilutions of the multidrug-resistant, Pgp-negative small-cell lung cancer cell line NCI-H1688 (50 and 25 $\mu$g) was used as the positive control. STD is the molecular weight standard lane.

three had marrow blasts with detectable P-95. Among the total 39 AML patients studied, no FAB subtype or cytogenetic abnormality correlated with P-95 expression.

*Correlation of P-95 Western blotting with dnr accumulation and retention*

Concomitant flow cytometric assays measuring 3 h accumulation of dnr (1 $\mu$g ml$^{-1}$) were performed on AML blast cells from the 46 patient marrows used for P-95 Western blotting. Sixteen of these 46 samples were positive for P-95 expression by Western blotting. Thirty-seven of the 46 samples also had flow cytometric assays of dnr retention performed 16 h after washing the blast samples free of extracellular drug. Of the 37 marrow samples tested for dnr retention, 13 were positive for P-95 by Western blotting.

The P-95-positive marrows had reduced accumulation of dnr relative to the negative marrows (Figure 2). The difference was statistically significant using a Mann–Whitney test, with $P = 0.033$. Only one of the P-95-positive samples had an accumulation value greater than the median of the P-95-negative samples. A trend was also noted for decreased dnr retention in the P-95-positive marrows (Figure 2), with $P = 0.067$. There were no P-95-positive samples with high retention of daunorubicin.

All of the AML marrows described above had dnr accumulation and retention measurements performed with or without the addition of 5 $\mu$M cyclosporin A. Forty-three samples (27 P-95-negative, 16 P-95-positive) also had dnr accumulation experiments performed with or without the addition of 6.6 $\mu$M verapamil. Thirty-four samples (21 P-95-negative and 13 P-95-positive) had dnr retention experiments performed with or without verapamil. No significant difference between the P-95-positive and -negative groups with respect to the percentage enhancement of dnr accumulation or retention by verapamil or cyclosporin A was noted. This was true for the whole group, as well as for the subset of samples from previously untreated AML patients. Verapamil or cyclosporin A caused a greater than 20% enhancement of dnr accumulation in more than 25% and 50% of these patients respectively; however, this enhancement did not correlate with P-95 status.

*Dnr accumulation and retention in breast cancer sublines*

To examine further the association between P-95 expression and facilitated export of dnr, we performed dnr accumulation

**Figure 2** Correlation of dnr accumulation and retention, in AML blast cells, with P-95 expression by immunoblotting. The intracellular dnr content of the blast cells was quantitated by a flow cytometric measurement of dnr fluorescence. The amount of intracellular dnr accumulated immediately following a 3 h incubation with 1 μg $ml^{-1}$ dnr as well as the amount of drug retained 16 h after washing the cells free of external drug were determined. The median values for dnr accumulation and retention are shown by horizontal bars.

**Figure 3** Dnr accumulation and retention, measured in the P-95-positive drug-resistant breast cancer subline, MCF-7/AdrVp (▲), and its drug-sensitive parental cell line MCF-7/W (X). Cells were exposed to dnr (1 μg $ml^{-1}$) for varying periods of time. Flasks were either assayed immediately for intracellular dnr content or washed in ice-cold PBS and placed in a prewarmed culture medium without dnr for varying times to measure the retention of the drug. The cells were detached with trypsin and intracellular dnr content was quantified by flow cytometry. The data points represent the means of two different experiments. The vertical bars represent the range.

and retention experiments in MCF-7 cells and in the MDR subline MCF-7/AdrVp, which overexpresses P-95 (Figure 3). An alteration in overall intracellular drug transport had not been previously reported in MCF-7/AdrVp cells. Both cell lines had measurements of dnr content for up to 3 h after being placed in 1 μg $ml^{-1}$ of the drug. As shown in Figure 3, the accumulation of dnr reached a plateau in both lines by 3 h, but the intracellular dnr content of the resistant MCF-7/AdrVp cells was far less than that of the sensitive parent cells. After washing the cells to remove extracellular dnr at 3 h, the retention of dnr at various time points was measured. As shown in Figure 3, the P-95-positive MCF-7/AdrVp cells rapidly lost almost all detectable dnr fluorescence. The sensitive MCF-7 cells, starting from a higher plateau of accumulated dnr, also showed a loss of dnr fluorescence after washing the cells free of extracellular drug, but the MCF-7 cells retain approximately 40% of the accumulated dnr 2 h after the wash. The alteration in drug accumulation observed was not due to differences in cell size between the sensitive and drug-resistant sublines. Coulter volumes were found to be 2,468 ± 116 $\mu m^3$ for MCF-7/AdrVp and 2,683 ± 100 $\mu m^3$ for MCF-7 cells.

A small but consistent enhancement of dnr accumulation by cyclosporin A was noted in the P-95-positive MCF-7/AdrVp subline (Figure 4). No enhancement of dnr accumulation by cyclosporin A was noted in the drug-sensitive MCF-7 parent line. The approximate 40% enhancement of dnr accumulation was noted at 3 μM cyclosporin A, and was maintained at concentrations between 3 and 30 μM.

**Figure 4** Percentage change in dnr accumulation in MCF-7/AdrVP (□) and MCF-7 (■) cells by co-incubation with cyclosporin A. Cells were exposed to dnr (1 μg $ml^{-1}$) with cyclosporin A, at concentrations from 0 to 30 μM, for 3 h Intracellular dnr content was determined by flow cytometry, as described in the Materials and methods section. The data shown represent the means of two identical experiments, performed in triplicate on different days. The vertical bars represent the range.

### *Correlation of P-95 expression with dnr cytotoxicity*

Forty-one AML blast samples were examined for both P-95 expression and *in vitro* sensitivity to dnr. Western blot analysis demonstrated 26 of these samples to be P-95 negative and 15 to be P-95 positive. No statistically significant difference, overall, was found between P-95-positive and P-95-negative samples with respect to dnr cytotoxicity (Figure 5), although the difference in median cell survival between P-95-positive and -negative samples exposed to 1.0 μM dnr reaches statistical significance, with $P = 0.05$.

The same 41 AML marrow specimens were also tested for dnr cytotoxicity in the presence of 5 μM cyclosporin A or 6.6 μM verapamil. Greater than 40% enhancement of dnr cell killing by either cyclosporin A or verapamil had previously been observed in more than 60% of the samples studied (Ross *et al.*, 1993). No significant difference in enhancement of dnr cytotoxicity with either modulator between the P-95-positive and -negative groups was noted.

### *Western blotting for Pgp*

Thirty-four AML marrow lysates, from 25 patients, for which P-95 Western blotting was performed, also had immunoblotting performed for Pgp with the C219 monoclonal antibody (Ross *et al.*, 1993). Nine patients were studied twice. None of the 34 AML lysates had detectable Pgp expression, although clearly positive expression of Pgp in the DC3F/ADX multidrug-resistant control line was seen with each blot (data not shown). A Pgp band was even noted in the drug-sensitive parental DC3F cells, demonstrating the sensitivity of the assay. Nine of these 25 patients had AML

**Figure 5** Percentage of AML cells surviving *in vitro* culture with varying concentrations of dnr, analysed in relation to expression of P-95. Each marrow sample was subdivided, and exposed to either 0, 0.1, 0.3 or 10 µM dnr. The percentage of viable cells in each group was calculated using the FDA/PI flow cytomeric cell survival assay, as described in the Materials and methods section. The number of cells from each sample surviving with no dnr was set at 100%. The horizontal bars denote the median percentage of survival of P-95-positive and -negative AML samples at each dnr concentration.

blasts positive for P-95. Among an additional 13 AML lysates, for which P-95 was not tested, three samples had detectable Pgp (Ross *et al*., 1993).

*Association of P-95 expression with clinical response*

Of the 29 AML patients who were previously untreated with cytotoxic chemotherapy, four patients with acute proganulocytic leukaemia were not evaluated for clinical response because they were treated with all-*trans* retinoic acid. Of the 25 patients treated with daunorubicin (45 mg $m^{-2}$ $day^{-1}$ for 3 days) and cytarabine (200 mg $m^{-2}$ $day^{-1}$ for 7 days), 16 were P-95 negative and nine were P-95 positive. Ten of the 16 P-95-negative patients (63%) and five of the nine P-95-positive patients (56%) achieved complete remissions, suggesting that P-95 expression and clinical response are independent ($\chi^2 = 0.116$, $P$ = NS) in patients treated with the combination of cytarabine and daunorubicin.

### Discussion

This exploratory study demonstrates that the surface membrane-resident P-95 protein, originally found to be overexpressed in breast cancer cells made resistant *in vitro* to doxorubicin, is present on and may be associated with lower accumulation of dnr in leukaemic blast cells. This is not likely to be due to concurrent Pgp expression since only three of the AML lysates from our overall cohort of 49 previously untreated patients and 0/34 of our current samples had detectable Pgp by Western blotting (Ross *et al*., 1993). The MDR breast cancer subline, MCF-7/AdrVp, which overexpresses P-95, also has decreased accumulation and retention of dnr relative to the drug-sensitive parental cell line. This is the first demonstration that P-95 is associated with altered drug transport, both in the resistant cell line and in clinical samples. These studies suggest that P-95 may have an efflux transporter function and may represent another mechanism responsible for anthracycline resistance in human neoplasms.

The MCF-7/AdrVp subline has previously been shown to have no detectable *mdr1* transcription by Northern blotting (Chen *et al*., 1990), and we have found that *mdr1* expression in this line was undetectable by a reverse transcriptase–PCR assay (data not shown). The MCF-7/AdrVp subline has only a minor decrease in topoisomerase II expression relative to the MCF-7 parent line (Chen *et al*., 1990), and recent studies have demonstrated that topoisomerase II expression in AML blast cell specimens does not directly correlate with clinical response to induction chemotherapy (Doyle *et al*., 1992; Kaufman *et al*., 1994). While expression of the P-95 membrane protein has not been proven to cause low dnr accumulation in MCF-7/AdrVp cells, it links this subline with a subset of AML blast samples which similarly express P-95 and have low dnr accumulation.

AML cells expressing P-95 did not have greater enhancement of anthracycline accumulation than P-95-negative samples after exposure to MDR modulators such as verapamil or cyclosporin A. This is consistent with our finding that the enhancement of dnr accumulation in the P-95-positive MCF-7/AdrVp cells in response to CsA was relatively small compared with classic Pgp-expressing cell lines such as HL-60/Vinc, which in our studies had increases of up to 700% in anthracycline accumulation and retention (Ross *et al*., 1993). The blast cell accumulation and retention studies are consistent with our *in vitro* cytotoxicity studies, which indicate that AML blasts expressing P-95 have a trend towards greater survival after exposure to daunorubicin, yet have no statistically significant enhancement of dnr cytotoxicity by verapamil or CsA relative to P-95-negative cells.

A number of other membrane proteins have been associated with drug resistance. For example, an 85 kDa membrane protein, identified by a monoclonal antibody MRK20, has been reported as a marker for doxorubicin resistance (Hamada *et al*., 1988). The cDNA encoding this protein has recently been cloned and found to be identical to CD36, a cell-surface adhesion molecule of endothelium, platelets and monocytes (Sugimoto *et al*., 1993). Studies using the MRK20 antibody to examine the MCF-7/AdrVp cell line did not detect expression of the 85 kDa protein (Chen *et al*., 1990). Examination of the doxorubicin-resistant ovarian cell line 2780Ad, which expresses the 85 kDa protein, with antisera against P-95 did not reveal any expression of the P-95 antigen (Chen *et al*., 1990). We have recently screened MCF-7 and MCF-7/AdrVp cells with VM58, another monoclonal antibody specific for CD36, using an indirect immunofluorescence assay. Neither line was reactive with VM58, making it unlikely that CD36 and P-95 are the same protein.

A Pgp-negative MDR leukaemia subline HL-60/Adr, selected for resistance to doxorubicin, has been reported to overexpress a 190–195 kDa membrane protein compared with the sensitive parent line (McGrath *et al*., 1989). This protein is identical to the product of a gene amplified and overexpressed in a MDR small-cell lung cancer subline H69AR (Cole *et al*., 1992; Krishnamachary and Center, 1993). This recently cloned gene codes for a 190–195 kDa protein termed the multidrug resistance-associated protein (MRP). Sequence analysis reveals that MRP is a member of the ATP-binding cassette (ABC) superfamily of transport systems (Higgins *et al*., 1992). The relation of P-95 to MRP in mediating non-Pgp MDR in leukaemia, lung cancer and other neoplasms is unknown, although we find that P-95 is not overexpressed in the HL60/Adr cell line, and reverse transcriptase–PCR assays do not demonstrate MRP overexpression in the MCF-7/AdrVp cells (unpublished observation).

Bone marrow aspirates from two of our relapsing AML patients demonstrated higher P-95 expression than did aspirates from the same patients prior to chemotherapy. These findings, coupled with the initial observation of P-95 expression in breast tumour recurrent after doxorubicin-based chemotherapy, suggest that cancer cells expressing P-95 may be selected for *in vivo* by the administration of chemotherapy (Chen *et al*., 1990). While our study concentrated on AML, we have found that three patients with acute lymphoblastic leukaemia who had a poor response to induction chemotherapy also expressed high levels of P-95 on their lymphoblasts. We have recently found that membrane staining of P-95 protein can be detected by immunohistochemical techniques in resistant lung cancer and breast cancer cells which have been fixed in paraffin. The preservation of antigenic determinants of P-95 in fixed material should allow screening of solid tumour specimens, to correlate P-95 expression with response to chemotherapy and patient survival.

The broad 95 kDa band detected by the antisera appears to be a characteristic of the protein itself and not an artifact of the electrophoretic technique. Rehybridisation of the blots with antisera specific for topoisomerase II or actin revealed the expected sharp bands (data not shown). Incubation of NCI-H1688 cells in tunicamycin or cleavage of its membrane proteins with Peptide N-glycosidase F (PNGase F) causes diminution of the broad P-95 band and the appearance of a sharp 35 kDa band reactive with anti-P-95 antisera (manuscript in preparation). This 35 kDa band is presumably the peptide core of the P-95 glycoprotein.

Lysates from the multidrug-resistant lung cancer line NCI-H1688, hybridised with the P-95 antisera, also had a cross-reacting high molecular weight bands of unknown significance, as shown in Figure 1. This high molecular weight band in NCI-H1688, but not MCF-7/AdrVp, is demonstrable in three different antisera against P-95, generated against either MCF-7/AdrVp or NCI-H1688 membranes. The high molecular weight protein does not appear to be P-glycoprotein or MRP since the genes encoding these proteins are not detectably overexpressed in NCI-H1688 or MCF-7/AdrVp cells by reverse transcriptase PCR assays. A lack of total specificity of the P-95 antisera is not surprising, since the gel-purified 95 kDa band is the best current immunogen, but the 95 kDa band by Western blotting can reliably distinguish the drug-resistant cell lines NCI-H1688 and MCF-7/AdrVp from drug-sensitive MCF-7 cells.

In AML, at present, we have not seen an obvious correlation between P-95 expression and clinical response to initial induction chemotherapy, but this may be because of the small number of patients, tumour heterogeneity, the contribution of cytarabine to the regimen and the possible contribution of other mechanisms of resistance such as those described above. Based on the association of P-95 expression with decreased accumulation of daunorubicin in AML cells, however, we feel that P-95 may have a role as a mediator of anthracycline resistance in some AML cells.

The association of P-95 with decreased dnr retention, as well as accumulation, and the small modulatory effects of CsA are more obvious in the MCF-7/AdrVp subline than in AML blasts. These differences may be due to the difficulties in comparing a subcloned cell line, made drug resistant *in vitro*, with a heterogeneous population of unselected leukaemia cells in a clinical specimen. Immunohistochemical studies have shown tremendous heterogeneity of P-glycoprotein and topoisomerase II expression in AML blasts (Kaufman *et al.*, 1994). It is very likely that AML samples that are positive for P-95 by Western blotting will still have a large proportion of cells that do not express the protein. More convincing evidence for the role of P-95 as a drug resistance protein will require cloning of the cDNA encoding P-95 and demonstrating decreased anthracycline accumulation and drug resistance in cells that have been transfected with the cDNA.

**Acknowledgements**
The authors wish to thank Dr Y Tong, Mr W Yang, Ms B O'Connell, Ms Y Gao, Ms P Wooten and Mr J Chin for expert technical assistance during these investigations. This study was supported by NIH Grant CA 52178, American Cancer Society Grant CN-58 and by the Bristol-Myers company, under a research grant programme for Studies of Tumor Cell Resistance to Chemotherapy.

**References**

CHEN Y-N, MICKLEY LA, SCHWARTZ AM, ACTON EM, HWANG H AND FOJO AT. (1990). Characterization of adriamycin-resistant human breast cancer cells which display overexpression of a novel resistance-related membrane protein. *J. Biol. Chem.*, **265**, 10073-10080.

COLE SPC, BHARDWAJ G, GERLACH JH, MACKIE JE, GRANT CE, ALMQUIST KC, STEWART AJ, KURZ EU, DUNCAN AMV AND DEELEY RG. (1992). Overexpression of a transporter gene in a multidrug-resistant human lung cancer cell line. *Science.*, **258**, 1650-1654.

DOYLE L, QIN JT, ROSS D, SRIDHARA R, MELERA P, LEE E AND SCHIFFER C. (1992). Expression of topoisomerase II in human leukemia cells (abstract). *Proc. Am. Assoc. Cancer Res.*, **33**, 237.

DOYLE LA, KAUFMANN SH, FOJO AT, BAILEY CL AND GAZDAR AF. (1993). A novel 95 kilodalton membrane polypeptide associated with lung cancer drug resistance. *Lung Cancer* **9**, 317-326.

FOJO AT, AKIYAMA SI, GOTTESMAN MM AND PASTAN I. (1985). Reduced drug accumulation in multiply drug-resistant human KB carcinoma cell lines. *Cancer Res.*, **45**, 3002-3007.

HALLIGAN BD, EDWARD KA AND LIU LF. (1985). Purification and characterization of a type II DNA topoisomerase from bovine calf thymus. *J. Biol. Chem.*, **260**, 2475-2482.

HAMADA H, OKOCHI E, WATANABE M, OH-HARA T, SUGIMOTO Y, KAWABATA H AND TSURUO T. (1988). $M_r$ 85,000 membrane protein specifically expressed in adriamycin-resistant human tumor cells. *Cancer Res.*, **48**, 7082-7087.

HIGGINS J, HYDE SC, MIMMACK MM, GILEADI U, GILL DR AND GALLAGHER MP. (1992). Binding protein-dependent transport systems. *J. Bioenerg. Biomembr.*, **22**, 571-591.

HWANG BD, SHYY S, CHEN A-Y, JUAN C-C AND WHANG-PENG J. (1989). Mutant KB cells with decreased EGF receptor expression: biochemical characterization. *Cancer Res.*, **49**, 958-962.

ITO Y, TANIMOTO M, KUMAZAWA T, OKUMURA M, MORISHIMA Y, OHNO R AND SAITO H. (1989). Increased P-glycoprotein expression and multidrug-resistant gene (mdr1) amplification are infrequently found in fresh acute leukemia cells. *Cancer*, **63**, 1534-1538.

JULIANO RL AND LING V. (1976). A surface glycoprotein modulating drug permeability in Chinese hamster ovary cell mutants. *Biochim. Biophys. Acta*, **455**, 152-162.

KARTNER N, EVERNDEN-PORELLE D, BRADLEY G AND LING V. (1985). Detection of P-glycoprotein in multidrug-resistant cell lines by monoclonal antibodies. *Nature*, **316**, 820-823.

KATO S, IDEGUCHI H, MUTA K, NISHIMURA J AND NEWATA H. (1991). Absence of correlation between cytotoxicity and drug transport by P-glycoprotein in clinical leukemia cells. *Eur. J. Hematol.*, **47**, 146-151.

KAUFMANN SH, KARP JE, JONES RJ, MILLER CB, SCHNEIDER E, ZWELLING LA, COWAN K, WENDEL K AND BURKE PJ. (1994). Topoisomerase II levels and drug sensitivity in adult acute myelogenous leukemia. *Blood*, **83**, 517-530.

KRISHNAMACHARY N AND CENTER MS. (1993). The MRP gene associated with a non-P-glycoprotein multidrug resistance encodes a 190-kDa membrane bound glycoprotein. *Cancer Res.*, **53**, 3658-3661.

MARIE J-P, ZITTOUN R AND SIKIC B. (1991). Multidrug resistance (mdr1) gene expression in adult acute leukemias: correlations with treatment outcome and in vitro drug sensitivity. *Blood*, **78**, 586-592.

MARQUARDT D, McCRONE S AND CENTER MS. (1990). Mechanisms of multidrug resistance in HL60 cells: detection of resistance associated proteins with antibodies against synthetic peptides that correspond to the deduced sequence of P-glycoprotein. *Cancer Res.*, **50**, 1426-1430.

McGRATH T, LATOUD C, ARNOLD ST, SAFA AR, FELSTED RL AND CENTER MS. (1989). Mechanisms of multidrug resistance in HL60 cells: analysis of resistance associated membrane proteins and levels of mdr gene expression. *Biochem. Pharmacol.*, **38**, 3611-3619.

NOONAN KE, BECK C, HOLZMAYER TA, CHIN JE, WUNDER JS, ANDRULIS IL, GAZDAR AF, WILLMAN CL, GRIFFITH B, VON HOFF DD AND RONINSON IB. (1991). Quantitative analysis of mdr1 gene expression in human tumors by polymerase chain reaction. *Proc. Natl Acad. Sci. USA*, **87**, 7160-7164.

ROSS DD, JONECKIS CC AND SCHIFFER CA. (1986). Effects of verapamil on in vitro intracellular accumulation and retention of daunorubicin in blast cells from patients with acute nonlymphocytic leukemia. *Blood*, **68**, 76-82.

ROSS DD, WOOTEN PJ, SRIDHARA R, ORDONEZ JV, LEE EJ AND SCHIFFER CA. (1993). Enhancement of daunorubicin accumulation, retention, and cytotoxicity by verapamil or cyclosporin A in blast cells from patients with previously untreated acute myeloid leukemia. *Blood*, **82**, 1288–1299.

ROTHENBERG M AND LING V. (1989). Multidrug resistance: molecular biology and clinical relevance. *J. Natl Cancer Inst.*, **81**, 907–913.

SATO H, PREISLER H, DAY R, RAZA A, LARSON R, BROWMAN G, GOLDBERG J, VOGLER R, GRUNWALD H, GOTTLIEB A, BENNETT J, GOTTESMAN M AND PASTAN I. (1990). MDR1 transcript levels as an indication of resistant disease in acute myelogenous leukemia. *Br. J. Haematol.*, **75**, 340–345.

SCOTTO KW, BIEDLER JL AND MELERA PW. (1986). Amplification and expression of genes associated with multidrug resistance in mammalian cells. *Science*, **232**, 751–755.

SUGIMOTO Y, HAMADA H, TSUKAHARA S, NOGUCHI K, YAMAGUCHI K, SATO M AND TSURUO T. (1993). Molecular cloning and characterization of the complementary DNA for the $M_r$ 85,000 protein overexpressed in adriamycin-resistant human tumor cells. *Cancer Res.*, **53**, 2538–2543.

TOWBIN H, STAEHELIN T AND GORDON J. (1979). Electrophoretic transfer of proteins from polyacrylamide gels to nitrocellulose sheets: procedure and some applications. *Proc. Natl Acad. Sci. USA*, **76**, 4350–4354.